# Perceptions regarding cardiovascular health and preparedness for digital health education among adolescents in an urban community of Nepal: A qualitative study

**DOI:** 10.1177/20552076251321068

**Published:** 2025-02-18

**Authors:** Dayana Shakya, Karin Flodin, Dip Raj Thapa, Madhusudan Subedi, Nawi Ng, Abhinav Vaidya, Natalia Oli, Alexandra Krettek

**Affiliations:** 1 Department of Internal Medicine and Clinical Nutrition, Institute of Medicine, 70712 Sahlgrenska Academy, University of Gothenburg, Gothenburg, Sweden; 2 Department of Nursing, 128550 Kathmandu Medical College, Kathmandu, Nepal; 3 Department of Public Health, School of Health Sciences, 7359 University of Skövde, Skövde, Sweden; 4 Department of Caring Sciences and Reproductive Health, Perinatal and Sexual Health, School of Health Sciences, 7359 University of Skövde, Skövde, Sweden; 5 School of Public Health, 415559 Patan Academy of Health Sciences, Lalitpur, Nepal; 6 School of Public Health and Community Medicine, Institute of Medicine, 70712 Sahlgrenska Academy, University of Gothenburg, Gothenburg, Sweden; 7 Department of Community Medicine, 128550 Kathmandu Medical College, Kathmandu, Nepal; 8 Department of Community Medicine, Faculty of Health Sciences, 8016 UiT The Arctic University of Norway, Tromsø, Norway

**Keywords:** Digital health education, cardiovascular disease, mobile games, adolescents, LMIC, qualitative

## Abstract

**Background:**

Cardiovascular disease (CVD) is the leading cause of death in Nepal. As CVD risks can develop early in life, a life course approach for non-communicable disease (NCD) prevention is needed. Due to its potentially acceptable delivery mode, digital health education could be a promising way forward to increase adolescents’ CVD knowledge.

**Purpose:**

The purpose of this study was to explore adolescents’ CVD perceptions and their perceptions and preparedness for digital cardiovascular health education through mobile games.

**Methods:**

Twelve focus group discussions were conducted with adolescents, Grades 8–10, from two public and four private Nepalese schools. A qualitative study with a deductive thematic analysis was performed, guided by the health belief model (HBM) and the technology acceptance model (TAM).

**Results:**

The analysis resulted in 6 themes and 13 sub-themes concerning perceptions of CVD and 5 themes and 10 sub-themes on perceptions and preparedness for digital cardiovascular health education through mobile games. The adolescents viewed CVD as a serious disease with consequences. A healthy diet and physical activity were important for prevention. Benefits were the positive impacts of a heart-healthy lifestyle. Barriers were the temptation of consuming unhealthy food, lack of healthy food environments, time and motivation. The adolescents also stressed their own ability to prevent CVD. Digital cardiovascular health education through mobile games was desirable. Constraints were accessibility and technical issues, parental allowance, available time and whether the game was engrossing enough.

**Conclusion:**

The adolescents perceived CVD as serious, with benefits and barriers connected to its prevention. Digital cardiovascular health education through mobile games was viewed positively but not without constraints for successful implementation.

## Introduction

Cardiovascular disease (CVD) is the leading cause of death and disability-adjusted life years (DALYs) in Nepal.^1^ In 2019, CVD contributed 24% of total deaths and 11.9% of total DALYs. The 2019 WHO STEPS survey identified a high prevalence of CVD risk factors in Nepal. In addition, 3.3% of adults aged 40–69 have a 10-year CVD risk of >30%.^2^ According to the latest Global School-based Student Health Survey (GSHS) in Nepal, a majority of children also eat less than the five servings of fruits and vegetables per day recommended by the World Health Organization (WHO) and are physically inactive.^3^ A study in a semi-urban area of Nepal determined that most children prefer unhealthier food items and exhibit low physical activity.^4^ Despite a higher prevalence of CVD as well as its risk factors, the knowledge, attitude and practice regarding CVD are also found to be poor in multiple studies.^5,6^

The risk for developing CVD starts as early as foetal life^7^ and can lead to cardiovascular events in adulthood.^8^ Lifestyle and habits related to diet and physical activity, which are crucial for cardiovascular health promotion, often form in early childhood.^9,10^ Hence, a life course approach is critical for efficient prevention and control of CVD.^11,12^

The government of Nepal, after facing challenges^13^ in the implementation of a multi-sectoral action plan (MSAP) for the prevention and control of non-communicable diseases (NCDs) 2014–2020, has formulated a second version. The MSAP 2021–2025 prioritises the equipment of local and provincial governments in their roles in the MSAP, utilising administrative restructuring to establish subnational coordination and engaging the non-state and private sectors in NCD interventions. Even though a life course approach to control NCDs is missing in Nepal, one proposed solution is to strengthen primary healthcare with the inclusion of such an approach.^14^

To fully apply a life course approach, health promotion for adolescents is an important focus area in low- and middle-income countries (LMICs) like Nepal, where prevention and control measures to date have largely focused on adults. School-based interventions are a productive way to effectively reach adolescents.^15,16^ Furthermore, efficiently reaching adolescents to increase their CVD awareness requires a medium that is both acceptable and user-friendly to this population.

The WHO ‘Best Buys’ focus on cost-effective and feasible interventions for NCDs. ‘Fit for the future’ is a project for adolescents launched by the Ministry of Health in Vietnam, with digital means for cardiovascular health promotion through schools.^17^ The American Heart Association (AHA) also emphasises using contemporary health education methods, such as mobile-based interventions and electronic gaming, to improve cardiovascular health in school children.^18,19^ AHA dietary guidelines can be incorporated into these digital interventions to reach a large population.^20^

Nevertheless, before planning such school-based interventions targeting CVD awareness, it is necessary to assess how adolescents perceive CVD^20^ and their perceptions and preparedness for digital cardiovascular health education through mobile games. Social and behavioural science theories are beneficial in guiding health promotion research by providing an encompassing view.^21^ The health belief model (HBM) can provide insight into perceptions about CVD that motivate changes in individual health behaviour. It is one of the most commonly used models for understanding health behaviour.^22^ To evaluate how adolescents perceive electronic media as a source of knowledge about CVD, the technology acceptance model (TAM) is useful as it relates to how different users can accept and use a novel technology.^23^

To date, there is limited information regarding adolescents’ knowledge and perceptions of CVD in Nepal and targeted preventive measures focusing on a life course approach are largely lacking.^14^ Studies on the implementation of digital health interventions highlight challenges such as inadequate technical infrastructure and opportunities related to the education and training of students.^24^ Mobile health interventions aimed at Nepalese adolescents’ sexual health education as well as adult Nepalese women's knowledge of maternal health, neonatal health and geohazards through a mobile game show promising results through increasing participants’ knowledge.^25,26^ However, especially in LMICs, there are limited qualitative studies regarding adolescents’ views and perceptions of digital health promotion interventions.^27^

The aim of this study was therefore to qualitatively explore adolescents’ perceptions regarding CVD and their perceptions and preparedness for digital cardiovascular health education through mobile games.

## Materials and methods

### Study design, setting and participants

We carried out a qualitative study with a postpositivist research perspective to explore contextualised insights and knowledge through the lived experiences of individuals.^28^ Focus group discussions (FGDs) were conducted among Grades 8–10 adolescents of the Jhaukhel-Duwakot Health Demographic Surveillance System (JD-HDSS), a semi-urban site in Nepal. The JD-HDSS was established by members of the research team in 2010 in Jhaukhel and Duwakot of Changunarayan Municipality in the Bhaktapur district of Nepal.^29^ Studies on CVD have been carried out in this area with an emphasis on adults.^4,30–35^ In contrast, this study focuses on adolescents. Ethical approval for this study was received from the Nepal Health Research Council (NHRC) (protocol registration number 660/2021, approval numbers 1828 and 862).

Of a total of two public and eight private schools up to Grade 10 in the JD-HDSS, we included two public and four private schools in the study. We used purposive sampling for school selection to ensure maximum variability in terms of tuition fee, geographic location, adolescent student numbers, experience of online classes and computer or internet facilities at school. The type of school can be taken as a proxy for socioeconomic status in Nepal. Parents with higher socioeconomic status prefer to enrol their children in private schools.^36^

Inclusion criteria consisted of girls and boys in Grades 8–10 studying at the selected schools of the JD-HDSS. Two FGDs—one for girls and one for boys—were conducted separately in each of the six schools, with a total of 12 FGDs. Consent was obtained from the parents to allow their adolescents to participate in the study, and only those with parental consent were included in the FGDs.

### Tools

The research team developed the FGD guides, based on the HBM and TAM theoretical models. We used the HBM to guide our focus on the perception of CVD and the TAM for perceptions and preparedness for digital cardiovascular health education through mobile games. The FGD guides were developed through an extensive literature review and consultation with the co-authors. The FGD guides developed were included in the ethical clearance by the NHRC. The FGD guides were pretested in two separate FGDs with girls and boys from one private school. The pretested FGDs were excluded from the final analysis. After pretesting, the sequence of questions was rearranged, and six probing questions were added to ensure an understanding of the questions.

### Theoretical framework

The HBM is used to explore knowledge and changes in health behaviour related to the individual. It consists of the key dimensions of perceived susceptibility, perceived severity, perceived benefits, perceived barriers, cues to action and self-efficacy.^22^ The TAM relates to how different users can accept and use a technology. It consists of the following dimensions: perceived usefulness, perceived ease of use, attitude towards using, behavioural intention to use and actual use.^23^

### Data collection

Permissions were obtained from the selected schools for inclusion in the study. Information letters were distributed to the adolescents and sent to their parents prior to the FGDs. Signed parental consent was collected from the adolescents during the following 2–3 days. The school principals were then contacted to decide on a feasible time for the adolescents to participate in the FGDs. The FGDs were carried out in separate rooms inside the school premises from 31 July to 15 December 2022. Only adolescents with parental consent were selected by the respective class teachers of Grades 8–10 to participate in the FGDs. Around three to four adolescents were selected from each class. Assent was taken from the adolescents before the FGD. Around 8–11 girls and boys were gathered for each separate FGD. The FGDs were conducted by researcher DS or a research assistant with a master's of science in nursing. A note taker with a bachelor of science in nursing assisted in the FGD documentation. The moderators and note taker were trained on conducting FGDs by the researcher MS. Researcher DS has previous experience conducting FGDs. The moderators for the FGDs were not related to the adolescents in any way that could have influenced the answers.

Each FGD lasted about 45 min to 1 h and was conducted without the presence of any schoolteacher to ensure that the students could talk freely. At the beginning of each FGD, the moderators established a good rapport with the participants to keep them comfortable sharing their experiences. This was done by explaining that whatever they shared would remain confidential and would not affect their grades. The objectives were also explained. A voice recorder was used with permission from the adolescents to record the FGD. After completing each FGD, the audio records were transcribed verbatim into Microsoft Word by a transcriber (with a bachelor's in sociology and a master's in rural development) and translated into English by a translator (with a master's in nursing). Researcher DS ensured the quality of the translations by cross-checking them with the transcriptions. The transcribed and translated versions of all FGDs were anonymised and stored with audio records on an external hard disc. We followed the project's data management plan for handling and storing data. The data were accessible only to the researchers.

Data were collected from 58 girls and 56 boys. Initially, two public and two private schools were selected and one FGD with girls and one with boys in each school was planned. Due to the heterogeneity of the groups and to achieve saturation, two additional private schools were added. This resulted in a total of two public and four private schools for the FGDs.

### Data analysis

We performed a thematic analysis using a deductive approach to generate the codes and sub-themes that formed the themes. A deductive approach was useful in this case, as the goal was to make use of existing theories about a phenomenon that would benefit from further description.^37,38^ We therefore applied the HBM and TAM to guide the analysis. The thematic analysis process consisted of the following steps: familiarisation with the data, code generation, determining, naming and reviewing themes and selecting applicable extracts to represent the themes.^39^

Two researchers (KF and DRT) analysed the 12 FGDs using manual coding for the transcribed texts. First, the transcribed texts were read and reread thoroughly by both researchers to make sense of the content and meaning of the data. In the next step, codes were developed by determining interesting data features that formed a coherent unit. Then, codes with similar meanings were grouped to form appropriately named sub-themes and abstracted to the appropriate theme for each model dimension. The identified themes and sub-themes were repeatedly checked against the data source. As a final step, all co-authors reviewed and refined the themes and sub-themes and verified them in relation to the presented extracts. An example of the coding process with the development of codes, sub-themes and themes is shown in Table 1.

**Table 1.** Example of the development of codes, sub-themes and themes.

Theme	Sub-theme	Code	Quote
Perceived susceptibility	Specific groups are prone to be affected by CVD	Older adults and children are at risk Those who are weak and elderly are at higher risk due to frailty Substance abusers and smokers are at risk	‘Old age people are at risk. It may also occur in children’ (FGD 3, private school, girls) ‘The weak ones and elderly people are at higher risk … because they are already weak’ (FGD 9, public school, girls) ‘… those who are substance abusers and smokers’ (FGD 10, public school, boys)
Perceived usefulness	Provide knowledge and awareness	Gain knowledge about what is good and bad for heart health Provide benefits to many as well as awareness Recognise a problem earlier through the knowledge gained by a game	‘It equips us with knowledge about what is good and what is bad for our heart's health’ (FGD 12, private school, boys) ‘It will be beneficial to many and create awareness about the disease’ (FGD 1, private school, girls) ‘If some problem occurs to us in the future, we can recognise it earlier, as we have learnt it via a game’ (FGD 10, public school, boys)

## Results

The final analysis resulted in 6 core themes and 13 sub-themes for adolescents’ perceptions regarding CVD and 5 core themes and 10 sub-themes for adolescents’ perceptions and preparedness for digital cardiovascular health education through mobile games. All themes and sub-themes are shown in Table 2.

**Table 2.** Overview of themes and sub-themes.

Theme	Sub-themes
HBM	
Perceived susceptibility	-Specific groups prone to be affected by CVD -Causes and preventive measures related to CVD
Perceived severity	-Different burdens of CVD -Impact of CVD on everyday life
Cues to action	-Raising awareness and increasing knowledge -Motivation through family, neighbours and home environment
Perceived benefits	-Health- and habit-related improvements -Improved living conditions
Perceived barriers	-Temptation of unhealthy food
	-Limited availability of healthy food environments
	-Limited time and motivation
Self-efficacy	-Own responsibility and ability to prevent CVD -Being dependent on others
TAM	
Perceived usefulness	-Provide knowledge and awareness -Attain information, creativity and entertainment
Perceived ease of use	-Easiness to learn and play games -Limited technical challenges
Attitude towards using	-Game features and design essential -Adventurous and challenging games preferable
Behavioural intention to use	-Realistic and useful games in daily life -Ability to play with friends and share games with family
Actual usage/preparedness	-Parental permission to play -Constraints to play

### Perceived susceptibility

Perceived susceptibility encompasses which persons could be affected by CVD, the specific reasons and the factors that cause and prevent it.

### Specific groups prone to be affected by CVD

Adolescents expressed that old age is a factor that contributes to the risk of developing CVD, even if they also thought that children could be affected. They also reasoned that those who are weak and older adults are at higher risk due to frailty. Participants stated that drug users, people who consume alcohol and smokers also have an increased risk of CVD.

Old age people are at risk. It may also occur in children. (FGD 3, private school, girls)

The weak ones and elderly people are at higher risk … because they are already weak. (FGD 9, public school, girls)

… those who are substance abusers and smokers. (FGD 10, public school, boys)

### Causes and preventive measures related to CVD

Several factors were perceived as causes of CVD, including physical, nutritional and environmental factors. The adolescents believe that a lack of exercise or a sedentary lifestyle causes CVD later in life. Similarly, food bought and eaten outside the home may cause it. The environment and pollution also increase the risk of developing CVD.

Sitting in one place using the mobile and a lack of exercise may contribute to the development of heart disease in the future. (FGD 3, private school, girls)

Food from the outside or street foods like instant noodles, biscuits and panipuri may cause heart disease. (FGD 9, public school, girls)

For example, the dust and smoke from factories and industries also affect our hearts. (FGD 9, public school, girls)

The preventive measures that were mentioned included practising heart-healthy habits and avoiding unhealthy ones. Feasible habits included consuming healthy foods, such as vegetables, avoiding unhealthy foods, such as fatty foods, and exercising to prevent disease. The adolescents also mentioned that it was important to perform frequent health check-ups and refrain from activities such as drinking alcohol and smoking.

We should eat foods that protect our bodies like green leafy vegetables. We should avoid junk foods, do regular physical exercise, yoga etc. Then …we can protect ourselves from diseases. (FGD 10, public school, boys)

Regular check-ups for heart disease and advice from doctors are necessary. (FGD 6, private school, boys)

Alcohol and smoking should be avoided. (FGD 8, public school, boys)

### Perceived severity

Perceived severity deals with how CVD can influence a person's daily life in various ways and the burdens that could affect a person.

### Different burdens of CVD

The adolescents mentioned several biological and physical burdens affecting a person with CVD. Some of these included problems with blood circulation. In addition, the person would be weak, experience symptoms of nausea and dizziness and could even fall due to low blood pressure.

Related to the heart … a lack of blood circulation due to any reason. (FGD 12, private school, boys)

People will fall. This can be in any place and at any time, because of low blood pressure. (FGD 7, public school, girls)

### Impact of CVD on everyday life

The impacts that adolescents raised as a result of acquiring CVD included that the financial status of the person would deteriorate, which would negatively affect the family. They also stressed that life would be worrisome and that there would be tensions in the family, with potentially detrimental consequences. Further impacts were that society might develop an unfavourable impression of the person with CVD.

He will not be able to earn money and support the family. (FGD 8, public school, boys)

There will be family problems. The family structure will weaken. (FGD 1, private school, girls)

The society's perspective will be different. The society might develop a negative attitude towards that person. (FGD 6, private school, boys)

### Cues to action

Cues to action deal with aspects that could influence and motivate a person to avoid developing CVD. This includes the importance of education and making people aware as well as how people and their surroundings could motivate them.

#### Raising awareness and increasing knowledge

The participants reasoned that raising awareness and sharing knowledge by educating people about what happens during CVD are essential. This was especially important among older adults. They also emphasised that awareness could be increased through different media.

Creating awareness is necessary … creating awareness and understanding about heart disease among old aged people. (FGD 12, private school, boys)

Social media might also help to create awareness. (FGD 5, private school, girls)

#### Motivation through family, neighbours and home environment

Other sources of motivation include personal connections and relations. The adolescents reasoned that if someone in the family or in the neighbourhood became affected by CVD, that could motivate a person to live a healthier lifestyle. In addition, an awareness of the causes and fear of suffering from the disease could motivate a person. The participants also pondered that the home environment was important. They felt that healthy eating habits came from home.

When someone at home is affected, you feel like you can also get it. (FGD 1, private school, girls)

… when we see others having a hard time because of illness. (FGD 11, private school, girls)

If everyone at home eats healthy foods, then I must also eat healthy foods. (FGD 5, private school, girls)

### Perceived benefits

Perceived benefits focus on how a person's life could be improved by adopting a heart-healthy lifestyle.

#### Health- and habit-related improvements

The adolescents mentioned that they perceived several benefits in relation to adopting heart-healthy habits. They reasoned that changes in habits and daily routines would be good for their health. Moreover, they considered that such changes meant that a person would remain disease-free or that it would be possible to prevent disease.

It brings a change in the daily routine. These healthy habits become like our activities of daily living. (FGD 6, private school, boys)

Chances of getting other diseases are reduced. It saves lives. (FGD 8, public school, boys)

#### Improved living conditions

The adolescents also envisioned that following heart-healthy habits would benefit people, as they could continue working and earn a living. In their view, it would improve their lives and allow them to gain financial and personal advantages. They thought it would encourage others to follow these people's examples.

If we are healthy, we can work until old age, we will be free from disease. (FGD 3, private school, girls)

We will remain free from heart disease. We won’t have any difficulty with our work. We can earn money and progress in life. (FGD 2, private school, boys)

It encourages others as well. Juniors will also follow you. (FGD 4, private school, boys)

### Perceived barriers

Perceived barriers entail those aspects that influence the person in not having a heart-healthy lifestyle.

#### Temptation of unhealthy food

The adolescents spoke about a desire to eat junk food after seeing advertisements in magazines or on television. Some participants also mentioned that there was more of an attraction towards junk food due to its look and taste.

After seeing an advertisement on television, we get a temptation to eat those foods. (FGD 8, public school, boys)

Because of its good taste, we prefer junk food over others. I can’t control myself to avoid those foods. (FGD 9, public school, girls)

#### Limited availability of healthy food environments

Others also emphasised that the absence of a canteen at school or the unavailability of a place to eat healthy foods caused problems in accessing healthy foods, leaving them with a lack of choices.

It is obvious to eat those foods when there is no canteen at school. If a canteen was available, probably, we wouldn’t have to take that much junk food. (FGD 8, public school, boys)

We don’t eat much, but the school's canteen … there are not many options left. That is why we consume junk … there are no options. (FGD 7, public school, girls)

#### Limited time and motivation

The adolescents reasoned around different circumstances that could cause barriers to heart health. This included a lack of time to prepare the desired food, which forces the adolescents to eat outside the home. Some mentioned that personal motivation is also an important factor. This was connected to physical activity, and spending more time on the mobile or watching TV was sometimes easier than doing exercise.

When the family doesn’t have enough time to prepare snacks, then we have to eat outside. (FGD 7, public school, girls)

We spend more time on the mobile and TV, and don’t want to be physically active. (FGD 1, private school, girls)

### Self-efficacy

Self-efficacy concerns adolescents’ reasoning about whether they feel they have the ability and confidence to prevent CVD and the different aspects that would influence whether they would or would not develop CVD.

#### Own responsibility and ability to prevent CVD

Some adolescents felt that there was a 50% risk of developing CVD for themselves and others that there was a 99% chance of them not developing CVD. This depended upon their ability to prevent CVD by being vigilant when it comes to food and performing daily exercise. Some stated that they would try their best but that they might develop CVD due to unhealthy food, decreased or no exercise or heredity.

No, there is a 99% chance of not getting heart disease … because we pay attention to our food, do daily exercise and eat a balanced diet. (FGD 8, public school, boys)

There is a chance … because of junk food, lack of exercise and hereditary predisposition. (FGD 4, private school, boys)

#### Being dependent on others

Some adolescents felt that not only do they find it difficult to adhere to heart-healthy habits, but they are also dependent on others or on circumstances that they cannot avoid. One participant mentioned that when they go outside, there can be someone sitting nearby smoking. This may affect the adolescent's health but is out of their control. Some also stated that they were left with no choice and had to eat outside the home due to their busy parents.

While going outdoors if someone sitting nearby smokes, it may affect us. (FGD 8, public school, boys)

My mother can’t prepare food, as she is busy … so I have to eat outside. That's why I am left with no option. (FGD 7, public school, girls)

### Perceived usefulness

Perceived usefulness revolves around the ways in which digital cardiovascular health education in the form of a mobile game could be considered useful for adolescents.

#### Provide knowledge and awareness

The adolescents felt that they would prefer gaining knowledge through a game about heart-healthy and unhealthy habits. They also perceived that a mobile game for cardiovascular health would help them and others become more aware of the disease. Some participants expressed that if they were affected in the future, they would know what to do due to gaining previous knowledge through a game.

It equips us with knowledge about what is good and what is bad for our heart's health. (FGD 12, private school, boys)

It will be beneficial to many and create awareness about the disease. (FGD 1, private school, girls)

If some problem occurs to us in the future, we can recognise it earlier, as we have learnt it via a game. (FGD 10, public school, boys)

#### Attain information, creativity and entertainment

The adolescents stated that it might be of interest to play a game to learn about CVD. Furthermore, the participants spoke about how a person would become creative and how it would help their ability to focus. In addition, some mentioned that such a game would provide both information and entertainment.

If the game is educational and informative, I might be interested. (FGD 4, private school, boys)

We can be creative by playing games. (FGD 2, private school, boys)

… will get information along with entertainment. (FGD 1, private school, girls)

### Perceived ease of use

Perceived ease of use concerns ease of learning and playing games. It also involves limitations, such as the need for limited technical challenges to play games.

#### Easiness to learn and play games

Some adolescents stated that games make things easy to understand and that they considered it easy to learn about CVD by playing a game. Some thought it depended on the game and upon the habit of playing games and that it could be difficult at first but not after getting accustomed to it. Others felt that it depended on the person, whether they found it easy or difficult to play the game.

… if such games can be played on the mobile phone, then we can easily learn things and it will be useful. (FGD 5, private school, girls)

It will be difficult in the beginning, but when we get used to it, then it won’t be so. (FGD 8, public school, boys)

… it depends on personal interest as well. (FGD 4, private school, boys)

#### Limited technical challenges

The adolescents also noted that technical challenges should be limited when playing games. This included access to the internet; the participants expressed concerns, such as if the power supply gets cut off or the internet speed goes down while playing the game. Some also voiced that it is necessary to have access to a mobile phone that is of good quality, as the kind of mobile affects the speed.

If the power supply gets cut off, only then will we have a problem. Otherwise, there won’t be any problem. (FGD 7, public school, girls)

One of the dominant factors is the mobile itself. The mobile affects the speed. It is good with a good phone but slow in a small phone. (FGD 8, public school, boys)

### Attitude towards using

The attitude towards using an educational mobile game involves aspects that are considered important and desirable in making adolescents want to play the game.

#### Game features and design essential

The adolescents mentioned the importance of game features and design. They also stressed that the game needed good graphics and animation. The features should be similar to other games and attractive with likeable characters.

The design must be appealing. The design of the game is of main importance. (FGD 4, private school, boys)

While opening the game … the game should attract us … like the feeling or desire to play. (FGD private school, girls)

I would find it interesting and enjoyable because of the characters inside it. (FGD 9, public school, girls)

#### Adventurous and challenging games preferable

The adolescents stressed that the game should be adventurous and should have increasing levels while playing. They also thought that a game that was competitive and had rewards would be fun. Some also stated that the game should have a mission or provide the possibility of completing tasks.

It will be more interesting if it is like an adventure game with increasing levels. (FGD 1, private school, girls)

If there are rewards and it is competitive, it is entertaining. (FGD 2, private school, boys)

The game must have a task that needs to be completed. (FGD 12, private school, boys)

### Behavioural intention to use

The behavioural intention to use educational mobile games involves the aspects that would influence adolescents to want to play the game.

#### Realistic and useful games in daily life

Adolescents emphasised that playing the game should feel real and motivate the person to become healthier. Some also stated that if the game were realistic, they would want to use it in their daily lives. Several adolescents felt that if a game was developed about heart-healthy habits, they would follow them.

If a character is real and performs real, I will copy it. (FGD 11, private school, girls)

It is not just about playing the game, but you should be able to follow it practically in real life. (FGD 8, public school, boys)

It must be interesting and should motivate us to follow healthy heart habits. (FGD 4, private school, boys)

#### Ability to play with friends and share games with family

The adolescents stated that they would prefer to play with friends. They also mentioned that if it were possible, they would prefer an offline game that they could play alone and an online version where they could collaborate as a group. The adolescents perceived it as positive if it was possible to share the game with the family.

The game should be feasible to play in a group among friends. (FGD 10, public school, boys)

It would be better if you could make a game that can be played offline while single and online while playing in a group. (FGD 6, private school, boys)

We can share the knowledge with family. (FGD 1, private school, girls)

### Actual usage or preparedness

Actual usage and preparedness concern those aspects that influence adolescents in having the actual ability and possibility to play such a game.

### Parental permission to play

Adolescents expressed that they would be very interested in playing but that it depended on the parents’ permission. They stated that their parents would allow them to play if it were an educational game that could be seen as beneficial to their studies. Some felt that it would be important to stress the usefulness of the game in learning about cardiovascular health so that parents would not think that it was like any other game.

At first, when parents hear the word game … they will say, what do you do with the game? What is the implication of playing the game? Then, when we convince them that this is an educational game and we gain knowledge through this, they will allow us to play. (FGD 8 public school, boys)

They will allow it if the game is related to heart disease, its preventive factors and if it provides information related to heart disease. If it is just like other games, then they will not allow us to play. (FGD 4, private school, boys)

The game should be such that family members should also support it. (FGD 4, private school, boys)

#### Constraints to play

Constraints that the adolescents voiced included that it was important not to have the game interrupted by different advertisements and pop-ups, such as in commercial games. They also mentioned that a game about cardiovascular health and healthy eating habits may not be interesting to everyone. Other concerns related to the fact that they had to borrow phones from their parents when the parents also needed them. They also stressed that they do not have much leisure time to play due to their studies.

No advertisement should appear in the middle of the game. The game should be advertisement-free. (FGD 6, private school, boys)

Shooting in a game is cool, but eating fruits and vegetables is not cool. (FGD 2, private school, boys)

Sometimes it will be difficult when we need the mobile at the same time as our parents need it. (FGD 8, public school, boys)

We do not have time as we have lots of homework to do. (FGD 7, public school, girls)

## Discussion

We found that the adolescents had a general understanding of CVD as a serious condition affecting a variety of persons with many biological and physical consequences. They perceived several ways to decrease disease risk. Risk-reducing behaviours are mainly related to adopting preventive CVD measures, including a healthy diet and physical activity, as well as knowledge, awareness and motivation. Benefits included positive individual impacts of a heart-healthy lifestyle. Barriers related to the temptation of unhealthy food, lack of healthy food environments, time and motivation. The adolescents stressed the importance of their own ability to prevent CVD and other people's influence on this practice. The adolescents also raised the importance of increasing knowledge and awareness regarding CVD and that digital cardiovascular health education through mobile games could contribute to increasing such knowledge. Usefulness, ease of use and appearance were important aspects of such games. Constraints revolved around accessibility and technical issues, time, parental allowance and whether the game was engrossing enough.

The increasing burden of CVD in Nepal has led to the need to establish targeted preventive measures and early interventions in primary healthcare. Research on CVD perceptions and perceptions and preparedness for digital cardiovascular health education through mobile games can help to focus on adopting a life course approach with the goal of rapidly reaching a larger population.^14^ In addition, an increased focus on digital health promotion in LMICs and especially the views of adolescents on such interventions to determine their utility for this age group is important.^27^

### Using the HBM and TAM as theoretical guidance

The HBM has been found useful in studies on adolescents regarding NCD risk factors in LMICs^40,41^ involving insight into knowledge and perceptions about different NCDs^42^ and particularly CVD.^43^ A limited number of such studies have been performed on CVD in adults in Nepal^44^ and not yet on adolescents. The TAM has been used to explore acceptance of technology regarding NCDs,^45^ but here too studies on adolescents and CVD are lacking. Only a few studies have used both the HBM and TAM as guidance quantitatively, but not yet on adolescents and CVD.^46–48^ Our dual approach of both determining adolescents’ CVD perceptions and perceptions and preparedness for digital cardiovascular health education through mobile games, using both models qualitatively for our research and data analysis process, was useful. To our knowledge, this is the first qualitative study to use both the HBM and TAM as guiding theoretical models for such an intent.

### Perceptions of CVD

Our study participants considered CVD to be related to various causes and to affect several different groups of people. Not only was it regarded to be severe to the individual but also to have a large impact on the person's and the family's financial situation, causing considerable stress. According to our participants, such a scenario would likely contribute to society's view of the person deteriorating. These views concur with the findings of a recent study in Nepal emphasising the serious social and economic consequences of NCDs for both individuals and their family.^49^

Similar to other studies on NCD knowledge and risk factors among adolescents,^42^ the participants recognised the individual's own control over the risk of developing the disease, with specific ideas on preventive measures to lower this risk. Preventive measures included consuming leafy greens, avoiding fatty foods and refraining from drinking alcohol and smoking. Vital practices for heart-healthy behaviours also involved doing exercise as well as frequent health check-ups. Such behaviours could, long-term, also lead to personal benefits, including financial and personal advantages, by staying employed and disease-free in the minds of our participants.

However, the adolescents recognised that there were several barriers to maintaining a healthy lifestyle. These included the ready availability, frequent advertisement and presence of junk food. Obstacles for healthy eating include a preference for fried food in Nepali culture and higher prices of fruits and vegetables with affordability issues, particularly in rural areas.^50,51^ The cost of a healthy diet is estimated to exceed the average yearly income by over 70%, leading to low worldwide intake in LMICs.^52^ Participants, especially from public schools in our study, emphasised the lack of a canteen at school or the unavailability of a healthy eating environment as concerns. This highlights the need for enabling environments in the community and schools to promote healthy behaviours.^41^

The adolescents in our study also stated that healthy eating habits and healthy practices were influenced by their family situation, time constraints, habits at home and personal motivation. If no one is available or has time to cook food for the child to bring to school, outside food has to be eaten. Maternal diets and perceptions about eating patterns also play a role in food choices and intake of healthy foods.^35,53–55^ In the minds of our participants, if the home environment encourages eating healthy foods, it contributes to adhering to healthy lifestyle practices. For physical activity, the adolescents felt it was sometimes difficult to find motivation to be active and it was easier to spend more time on the mobile or TV. These views concur with the findings about decreasing trends in physical activity and increased sedentary behaviour and screen time among adolescents worldwide.^56^

Studies in both high-income countries (HICs) and LMICs have noted similar barriers to those stated to adopting heart-healthy behaviours.^40,43^ However, contrary to other studies from HICs, where adolescents of Grades 9–12 do not perceive themselves at risk for CVD^57^ or to have low risk due to no current heart health difficulties,^43^ many of the participants in our study were aware of the risks and their own responsibility and ability to prevent CVD. They also emphasised raising awareness and knowledge through education about CVD, for example, through social media. Research on Nepalese adults found that younger adults have a higher awareness about their ability to be affected by CVD, as their age group is exposed to technology, which creates opportunities to develop awareness through education.^44^ Awareness programmes are useful for CVD awareness,^44^ but regardless of this, as is also evident in the views of our study participants, perceived risk has to come from within the individual to affect health-related behaviour change.^41,44^

### Perceptions and preparedness for digital cardiovascular health education

The adolescents in our study perceived that digital cardiovascular health education, in the form of a mobile game, would be useful in several ways. This is especially true in providing knowledge and raising general awareness about CVD in a creative way. This is similar to findings on adolescents’ perceptions about NCDs and the use of game-based education to increase knowledge regarding disease and prevention strategies^42^ and the mobile as useful to attain learning.^58^

The adolescents also expressed that game design, including the complexity of the game and its ease of use with a sufficient level of challenges, could help maintain interest in the game. These findings echo other studies in which interventions through gamification have been found particularly useful for adolescents^53^ and attractive game features to this age group were stressed.^59,60^

However, the majority of adolescents in our study emphasised that the acceptability of playing the game was heavily connected to limited technical difficulties while playing. This involved whether the mobile phone was powerful enough or the internet stable enough. These are similar factors that have been raised in the context of other LMICs.^61,62^ In Nepal, uneven ownership and internet coverage exist between urban and rural areas where geographical factors relate to differences in socio-demographic characteristics.^63–65^

Regarding behavioural intent and preparedness for digital cardiovascular health education through mobile games, our study participants stressed that a realistic game that was useful in their daily lives and provided motivation for better heart-healthy habits, along with the ability to identify with the characters, was important. In addition, the ability to play the game with friends and share the game with their families was vital. These aspects have also been described in a systematic review exploring user engagement in digital games for health promotion.^60^

We found that specific constraints for actual use or ability to play are associated with individual motivation and interest in playing mobile games related to cardiovascular health education, in addition to the need to have access to one's own phone or be able to use a parent's phone to play the game. It is not uncommon for several family members to share mobile phones and digital devices to minimise costs in Nepal.^64,65^ Other influential factors were the parental permission and timing of school work,^66^ where the adolescents emphasised that their parents had to be assured about the game's usefulness for their studies and the time needed due to the intensity of studies in these grades.

### Strengths and limitations

FGDs provided participants with the possibility of discussing CVD and digital cardiovascular health education through mobile games with other adolescents of the same gender, which added to the comfort level in the group. Moreover, even if the FGD facilitators could be perceived as a presence of authority, this was ameliorated by them sitting at the same eye level as adolescents by communicating throughout the FGD duration, assuring that adolescents could speak freely and that what they expressed in the FGD was confidential.

Other strengths related to adhering to trustworthiness, which included credibility, dependability, confirmability and transferability in the qualitative research process and thematic analyses.^39,67^ Credibility was established by purposive sampling of girls and boys from public as well as private schools, including representative quotes and a coding example to determine data fit between participants’ views and researcher representation. In addition, two researchers independently coded the transcripts with sub-themes and themes, which were then discussed among all co-authors. Dependability was determined by an open dialogue in the development and refinement of sub-themes and themes and a documented analysis process.^67^ Confirmability was assured by visualising the findings and interpretations of the data to all co-authors. Finally, transferability to similar contexts was facilitated through a clear description of the study setting and how the data collection and process occurred.^67^

Limitations of our study include the fact that all grade levels were represented simultaneously in the FGDs, possibly making those in the lower grades feel less comfortable expressing their opinions in the presence of older adolescents. FGDs also do not allow for an in-depth investigation of individual experiences. Expressing opinions about heart-healthy behaviours and the barriers to following them may have been sensitive for the adolescents and could be influenced by a social desirability bias. However, this was ameliorated by using a non-judgemental stance, ensuring confidentiality and anonymising the data by allocating the participants with numbers.

Using a deductive approach in data analysis was beneficial as a structured way to code the results, where the analysed codes and sub-themes had to fit in under the existing themes of the different models. This could, however, also provide a limitation compared to an inductive approach, where the analysis process is more fluid, based solely on the emerging concepts. Using two applicable theoretical frameworks, such as the HBM and TAM, for guiding the analyses, however, added to gain an encompassing and theoretically supported view on the data, as our focus was both on the perceptions surrounding CVD as well as technology acceptance.

## Conclusion

Adolescents perceived CVD as serious, affecting a variety of persons and having many health consequences, but also as preventable. Healthy diet and physical activity as well as knowledge, awareness and motivation were considered important for the prevention of CVD. Benefits involved the positive impact of a heart-healthy lifestyle, while barriers related to the temptation of unhealthy food, lack of healthy food environments, time and motivation. The adolescents also stressed the importance of their own ability to prevent CVD and other people's influence on their adherence to heart-healthy habits. The use of digital cardiovascular health education through mobile games was mainly perceived positively. Suggestions for its application included usefulness, ease of use and appearance, whereas constraints included smooth internet services, parental permission, the time for schoolwork and whether the game was engrossing enough.
